# Calf morbidity, mortality, and management practices in dairy farms in Jimma City, Southwestern Ethiopia

**DOI:** 10.1186/s12917-023-03815-w

**Published:** 2023-11-28

**Authors:** Umer Mifta Ahmedin, Alula Alemayehu Assen

**Affiliations:** School of Veterinary Medicine, Wollo University, 1145, Dessie, Ethiopia

**Keywords:** Calves, Hazard ratio, Incidence, Morbidity, Mortality, Survival analysis

## Abstract

**Background:**

This research aims to determine the incidence of calf morbidity, mortality and its potential predisposing factors in the first six months of life. Morbidity and mortality of dairy calves are persistent problems for dairy farmers worldwide. For effective control and prevention programs on calf health, it is imperative to estimate the extent of calf morbidity and mortality, and associated risk factors. Although few studies have investigated the epidemiology of calf morbidity and mortality in Ethiopia, comprehensive information is scarce in this area.

**Methods:**

Data were collected through a cross-sectional survey and longitudinal follow-up on purposively selected dairy farms. A longitudinal study was conducted on 235 calves from birth to 6 months of age. Survival analysis methods using the Kaplan–Meier (K-M) method, and mixed effect Cox proportional hazard regression were employed to compute the life-to-event data on morbidity and mortality.

**Results:**

From the 235 calves studied, 53 morbidity and 15 mortality events were recorded. This gives an overall morbidity and mortality incidence rate of 55 per 100-calf 6-months at risk (risk rate of 42.07%) and 14 per 100-calf 6-months at risk (risk rate of 12.97%), respectively. Diarrhea (13.84%) followed by pneumonia (8.97%) were the most common diseases that occurred in calves, respectively. Similarly, diarrhea (33.3%) and pneumonia (26.7%) were the leading causes of death. Dam parity (p < 0.001) and pen cleaning (p < 0.001) were significant predictors of calf morbidity. Dam parity (p = 0.007), calving status (p = 0.005), pen cleaning (p = 0.04), and floor type (p = 0.001) of houses were significantly associated with mortality. The hazard of diarrhea was significantly associated with sex (p = 0.003), first colostrum feeding time (p = 0.028), pen cleaning (p = 0.010), and breeding method (p = 0.013).

**Conclusion:**

The rates of morbidity and mortality reported in the study were higher than the economically acceptable, also affecting the welfare of the animals. The risk factors found need due attention in the management practices of dairy calves in Ethiopia.

**Supplementary Information:**

The online version contains supplementary material available at 10.1186/s12917-023-03815-w.

## Background

The morbidity and mortality of dairy calves are persistent problems for dairy farmersthroughout the world [1]. In hot areas, excessive temperature and humidity favor the proliferation and spread of pathogens to milk-fed calves, which impair the health of newborn calves and thereby affect heifer replacement [2]. Heifer replacement markedly influences dairy production by selective culling of low-productive cows on a farm [2, 3 & 4]. Diseases of young animals are the result of a cumulative interaction between management, environmental factors, pathogens, and risk factors [1]. Diarrhea and pneumonia account for the majority of morbidity and mortality in calves [5]. Risk factors such as inadequate or absence of colostral immunity, overcrowding, poor hygiene status, naive immune system in newborn animals, stress, and nutrition status have been documented for morbidity and mortality of calves [6].

In Ethiopia, earlier studies were conducted to determine the prevalence, incidence, and risk factors associated with calf morbidity and mortality [7–9]. A wide range of calf morbidity (22–62%) and mortality (5–30%) have been reported from different regions of the country. However, these reports used a cross-sectional design that only provides the magnitude of morbidity or mortality and related risk factors at a defined point in time. As a result, calf mortality/morbidity is investigated as a two-level outcome variable distinguished by whether or not a calf is morbid/dead after acquiring the pathogen. This considers animal sickness/death occurring during a defined period that overlooks the continuousness of the morbidity/mortality and the exact time of sickness/death [10]. Time-to-event analysis (survival analysis) provides numerous advantages over standard regression techniques. Survival analysis considers the continuity of the event of interest (i.e., morbidity/mortality) and does not constrain the data analysis to a predefined time. For effective control and prevention programs on calf health, it is imperative to estimate the extent of calf morbidity and mortality and comprehend the causes, methods of transmission, and associated risk factors. In addition, the management practice of calves is also a key factor influencing their wellbeing. Thus, the information obtained from the dairy farms provides insights into farmers’ practices, perceptions, and priorities [11]. In this regard, there is a scarcity of information about the epidemiology of calf morbidity and mortality in Ethiopia. Hence, the objective of this study was to describe the calf management practices on Ethiopian dairy farms and to determine the extent of cumulative morbidity and mortality incidence and the potentially related risk factors in calves from birth until six months old.

## Methods

### Study area

This study was conducted in Jimma City, which is located 355 km away from the capital, Addis Ababa, Oromia Regional State, southwestern Ethiopia. Although the prevailing dairy production system in Ethiopia is rural (98%) with predominantly local Zebu cattle breeds, this study was conducted on dairy cows kept under urban and peri-urban commercial dairy production systems. The area is located at a latitude of 7°41’N, a longitude of 36°50’E, and an elevation of 1704 m above sea level (masl). The climatic condition is characterized by a humid tropical climate with heavy annual rainfall ranging from 1200 to 2000 mm per year. From the total annual rainfall records, approximately 70% is received during the rainy season, from the end of May to early September. The average annual maximum and minimum temperature ranged from 25°c to 30°c and 7°c-12°c, respectively. The presence of plentiful rainfall in the area makes it conducive to agriculture [12].

### Study farm and animals

Fifty-three dairy farms with herd sizes ranging from 5 to 140 lactating cows that were willing to participate in the study were selected from a total of 113 registered dairy farms in Jimma City, Southwest Ethiopia. Calves between 1 and 180 days old were the study’s target population. Ethiopia has been using a crossbreeding scheme to combine superior hardiness, heat tolerance, disease resistance, and environmental adaptability of indigenous cattle with superior high milk yield, faster growth rates, and early maturity of temperate breeds, mainly Holstein Friesian cattle breed. The farms were urban and peri-urban commercial dairy farms. Dairy farms with more than 20 dairy cattle were characterized as large-sized dairy farms, and those with less than 20 dairy cattle were grouped as small-scale [13]. Thus, 30 small and 23 large-scale dairy farms were considered during this study.

### Study design and sampling method

A cross-sectional survey was conducted using a semi-structured questionnaire to gather herd data related to calf morbidity and mortality and to study calf management practices in selected dairy farms. The questionnaire was pretested on 8 dairy farms from the target group to refine the questionnaire design and identify errors. Moreover, a prospective longitudinal study was employed to collect data on the morbidity and mortality of calves from birth to 6 months between March 2021 to August 2021.

The farms and calves were conveniently selected considering the willingness of farmers to keep complete registration records on death, diseases, and treatment and allow the use of farm data. In addition, farms with five or more cows were selected to maximize the probability of finding at least one calf for follow-up.

Calves with few birthdays (< 1 month of age) at the beginning of the study and whose disease history and date of birth known were recruited retrospectively. Other calves born in the subsequent months were enrolled prospectively for the study. Due to the lack of calf disease history, only calves born on the farm were considered, and those purchased or given as a gift were not included in the study. Based on these criteria, 235 calves were recruited to conduct the follow-up.

### Study methodology

#### Questionnaire study

Herd-level potential risk factors associated with calf morbidity and mortality were collected from the farms through a semi-structured questionnaire. The questionnaire was pretested and administered to farmers and/or farm attendants through one-on-one interviews. The willingness of respondents was sought, and they were made aware of the study’s content and objectives, as well as informed that their participation was voluntary and their identity would be kept confidential, and verbal consent was obtained from each respondent.

Variables were collected at the calf and herd level and other farm management practices that are believed to have an association with calf morbidity and mortality. Accordingly, information on farm characteristics, general management practices, and calf rearing (colostrum management, housing, feeding, and health) were collected. The questionnaire sample is attached under the supplementary material (10.6084/m9.figshare.19519828.v1).

#### Longitudinal study

A total of 235 dairy calves with a mean of 4.52 (SD = 1.42; min = 2 and max = 9) calves per farm were examined during the follow-up period. The calves were recognized by their identification number, if not by number of the mother’s ID. The calves from the selected farms were visited every 15 days by a veterinarian and a data collector until they reached 6 months of age. At each visit, the veterinarian clinically examined all calves born and recorded prevalent diseases not detected by the farmers in the health records. Health issues of the calves that occurred outside of the visits were communicated by dairy farmers to the veterinarian and data collector. Emergency visits were made when farms phoned to report a calf health problem. If a disease occurs, the calf was examined clinically to define the cause. The morbidity of a calf was defined as any disease condition with noticeable clinical signs that can eventually lead to death or permit therapeutic intervention during follow-up; whereas mortality was defined as any death incident that occurred on the calves irrespective of the cause. If a calf was sick or died, it was considered an uncensored observation. In contrast, if a calf was not sick or not dead, it was considered to be a censored observation, this also includes culling, selling, or loss [14, 15]. If a calf loss was encountered during the follow-up period, the date and cause of the loss were recorded. Thus, calves that became sick and/or died during the study period were uncensored because morbidity and mortality are the outcomes of interest. Hence, calves enrolled in the study were observed for about 12 times at most, unless losses because of sales or other reasons to follow up occurred. The average number of visits was 8.3 (SD = 3.9; min = 2, max = 12) times. The personnel in charge of recording events (i.e., the farm manager) was informed to notice and record any health issues and deaths that occurred between visits. The cause of morbidity and mortality was determined according to the standard case definition attached to the additional file legend collection (10.6084/m9.figshare.19519828.v1).

### Description of variables

#### Dependent (outcome) variable

The outcomes of the model were individual calf status for morbidity and mortality in the first six months of age. Additionally, the main causes of morbidity and mortality, diarrhea and pneumonia, are also outcomes of the model.

#### Explanatory variables

A total of 21 explanatory factors of calf morbidity and mortality were included: sex, breed, dam parity, calving status, dairy farm as a source of income, dairying experience, management system, herd size, breeding method, floor type, vaccination and prophylaxis of dam, calving pen, navel disinfection, first colostrum feeding time, methods of colostrum feeding, calf housing, calf pen bedding, calf pen cleaning frequency, amount of milk-fed and water provision. The classes of variables were stratified to enable the analysis and interpretation of results based on previous studies [9, 13, 16]. Cleaning the pen frequency was classified as regular/frequent cleaning (cleaning the pen with water and disinfectant every day) and irregular/infrequent cleaning (cleaning the pen with water and disinfectant any time of the week). Provision of water to the calves was assessed and categorized into ad libitum (freely available) and periodic (provision of water at a certain time interval of the day). Calving status was determined based on the farmers’ records; if the first or especially second stage of calving parturition was markedly prolonged for more than 6 h and required assistance was classified stated as dystocia as indicated by Noakes and Parkinson [17].

### Data management and analysis

The data on morbidity and mortality were stored in Microsoft Excel for data management and moved to R.v3.6.1 [18] for statistical analysis. Descriptive statistics using the percentage and frequency were used to describe the questionnaire survey. The morbidity and mortality rates were determined as incidence rates, which was the rate at which the event of interest appears per time at risk [19]. The incidence rate was computed using the formula in Eq. (1).

$$IR=\frac{Number of events occurs during observation period }{Total calf days at risk}\dots \dots \left(1\right)$$

Where, the numerator is the total number of events (morbidity or mortality) that occur in the study period, whereas the denominator is the overall calf days at risk from the commencement of the study until the follow-up period ends. For morbidity rate, calf days at risk were estimated from the start of the study until the calf displays a clinical sign of disease [16]. The time at risk for mortality was defined from the time the calf was recruited in the study until death occurs. Furthermore, the incidence rates calculated for morbidity and mortality were changed to risk rates using the formula recommended by Martin et al. [20]. The risk rate was computed using the formula in Eq. (2).

$$Risk rate=1-{e}^{\left(-true rate\right)}\dots \dots \left(2\right)$$

The median days, cumulative survival probability, and incidence of morbidity and mortality of calves from different causes were computed using the Kaplan-Meier model employing the package {survival} of R [21]. Moreover, K-M curves for the cumulative survival probability of the exposure factors were built using {GGally} package of R [22]. The survival probability was estimated by using the formula in Eq. (3)

$${S}_{t+1}={S}_{t}*\left(\frac{\left({N}_{t+1}-{D}_{t+1}\right)}{{N}_{t+1}}\right)\dots \dots \left(3\right)$$

Where S_t_ is the survival probability of the previous time interval t; N_t_ is the number of calves that are free from an event of interest and at risk of having the event at interval t, and D_t_ is the number of calves with the event of interest (morbidity/mortality) throughout interval t.

The relationship between the explanatory variables (fixed effects) and outcome variables (morbidity, mortality, diarrhea, and pneumonia) was determined by the mixed-effect Cox proportional hazard model [23]. The farm was included as a random effect to account for clustering. Each explanatory factor was assessed based on its crude relationship with outcome variables by the univariate mixed-effect Cox proportional hazard regression model. A cut-off value of 0.25 on the level of significance was used as the selection criteria for a variable to be included in the multivariable model. A multivariable mixed-effect Cox proportional hazards regression model was used to determine the effect of explanatory variables on calf morbidity and mortality. The final model was built using stepwise backward elimination of non-significant variables (p ≥ 0.05) for each outcome variable. The collinearity analysis to detect the overlapping predictor variables on the outcomes, and the variance inflation factor (VIF) *>* 10 or tolerance *<* 0.1 was a criterion of severe collinearity. Possible interactions between variables in the model were studied for biological relevance, but none was identified as plausible and no interaction terms were fitted in the model. The model assumption was taken into account and tested for any violation [24]. A p-value less than 0.05 was regarded to be significant. The hazard ratio coefficient estimates and its 95% CI were obtained for all variable in the final model. The mixed-effect Cox regression model used is presented in Eq. (4)

$$h\left(t\right)={h}_{0}\left(t\right){exp}^{{({\alpha }_{j} +\beta }_{1}{X}_{1}+{\beta }_{2}{X}_{2}+\dots {\beta }_{P}{X}_{P})}\dots \dots \left(4\right)$$

Where, h(t) is the expected hazard at time t, h_0_(t) is the baseline hazard, *X*_*1*_, *X*_*2*_, *X*_*p*_ are the predictors (or explanatory variables), *α*_*j*_ denotes the random effect associated with the *j*-^th^ herd/farm cluster and *β*_*1*_ + *β*_*2*_ +_…_. *β*_*p*_ are the coefficients for each explanatory variable.

## Results

### Description of dairy farms

#### Farm owner attributes

Women only owned 22.64% of farms, compared to men who owned 77.36% of farms. According to their educational status, 26.44% of the farm owners had completed their primary education, 24.52% their secondary education, 24.52% their college or university education, and 24.52% had no formal education. For 71.69% of the farms, milk production was the main source of revenue, but for 28.31% it was a secondary source. The majority (60.38%) of the farmers had at least five years of experience in the dairy industry, while 39.62% had fewer (Table 1).

**Table 1:** Frequency distribution of major management practices in the dairy farms (n = 53)

Variables	Categories	Frequency	Percentage
Management system	Intensive	48	90.56%
	Semi-intensive	5	9.44%
Breeding methods	AI	48	90.56%
	Natural service	5	9.44%
Floor type	Concrete	45	84.91%
	Soil	8	15.09%
Dam vaccination and prophylaxis	Not practiced	52	98.11%
	Practiced	1	1.89%
Calving pen	Absent	47	88.67%
	Present	6	11.33%
Navel disinfection	Not practiced	52	98.11%
	Practiced	1	1.89%
Colostrum importance	Yes	40	75.47%
	No	13	25.53%
First colostrum feeding time	Within 6 h	38	71.69%
	After 6 h	18	28.31%
Colostrum feeding method	Suckling	26	49.05%
	Bucket/hand feeding	28	50.95%
Calf housing	With dam	37	69.81%
	In separate house	16	30.19%
Bedding material in the calf pen	Absent	19	35.85%
	Present	34	64.15%
Calf pen cleaning Frequency	Regularly	35	66.03%
	Infrequently	18	33.97%
milk feed per day	≤ 3 L	38	71.69%
	> 3 L	15	28.31%
Water provision	Periodic	47	88.67%
	Free access	6	11.33%
Measures to treat sick calf	Calling professional	47	88.68%
	Take to clinic	3	5.66%
	Have employed professional	3	5.66%

^AI: Artificial insemination, Hr: Hour, L: Liter^

#### Farm characteristics

The management system of the farms were intensive (90.56%) and semi-intensive (9.44%) systems. The average herd size of dairy cattle per farm was 18.57 (SD = 6.52). The farms had both local and crossbred cattle. The study animals were both local zebu cattle (13) and crossbred (Holstein Friesian X zebu cattle) (222) calves. Most of the farms (90.56%) breed their animals using artificial insemination (AI), while the remaining (9.44%) employed bull as a breeding system. About 66.03% of the farms were cleaned the calf pen regularly, whereas 33.97% clean irregularly. The material used to build dairy barns in the farms was concrete (13.15%) and wood (86.85%). About 84.91% of the farms had concrete floors and the remaining (15.09%) had a soil floor.

#### Dry cow management and periparturient care

Except for one farm, all farms did not conduct pregnant dam immunization and prophylaxis. Most of the farms (86.84%) provided the cows with dry periods of less than or equal to 8 weeks, whereas 13.16% of the farms had a dry period of more than 8 weeks. Half of the respondents (50%) visit veterinarians during calving complications; however, the remaining customarily assist their cows. Navel disinfection was practiced only by one farm. The majority of the farms (88.67%) had no calving pen; instead, cows gave birth in the barn cubicle and walkway.

#### Colostrum management and feeding

About 75.47% of respondents were aware of the benefit of feeding colostrum to calves. on 71.69% of farms, colostrum was given to calves during the first 6 h of life, while the remaining were not awared the best time to give colostrum feed. The colostrum feeding methods were hand feeding (50.95%) and suckling from the cow (49.05%).

#### Calf housing

The calves were housed separately from cows in 69.81% of the farms and in the same barn as cows in 30.19% of dairy farms. Calves were separated from dams within 1–4 days after birth among 71.05% of the farms, while 28.95% farms separate immediately after birth.

#### Milk, dry feed, and water provision

The majority of the farms (71.69%) fed the calves less or equal to 3 L of cow milk per day and only 28.31% fed more than 3 L of milk per day. The age to introduce solid feed was between 1 and 2 weeks in 42.11% of farms while > 2 weeks in 57.89% of farms. Water provision was periodic in 88.67% of the farms.

#### Health care

Farmers called to veterinarian (88.68%) while the calves were sick; or else took the sick calf to veterinary clinic (17%). Only 5.66% of farms had employed animal health professioals. about 63.15% and 42.10% of farmers have mentioned calf morbidity and mortality are a major problem in their farms, respectively (Table 1).

### Longitudinal study

#### Calf morbidity and mortality rate

A total of 235 calves were monitored throughout the study period on 53 dairy farms to estimate the incidence of morbidity and mortality. A total of 53 (22.55%) cases of morbidity and 15 (6.38%) cases of mortality were noted among the study animals. All calves collectively contributed 19,334 and 17,469 calf days at risk, which is comparable to 107.41 and 97.05 calf 6 months at risk for mortality and morbidity, respectively. This results in an overall incidence rate of 55 cases per 100 calf 6 months at risk (risk rate of 42.07%) and 14 cases per 100 calf 6 months at risk (risk rate of 12.97%) for morbidity and mortality, respectively (Table 2).

**Table 2:** Incidence (incidence rate and risk rate) of crude calf morbidity and mortality and specific disease conditions

Description	N	Calf day at risk	Calf 6 months at risk	Incidence	
				Incidence rate/6 calf months at risk (95%CI)	Risk rate %
Crude calf morbidity	53	17,469	97.05	0.55 (0.41,0.71)	42.07
Crude calf mortality	15	19,334	107.41	0.14 (0.08,0.23)	12.97
Diarrhea	16	19,237	106.87	0.15 (0.09, 0.09)	13.84
Pneumonia	10	19,259	106.99	0.09 (0.05,0.17)	8.97
Septicemia	7	19,304	107.24	0.06 (0.03,0.13)	6.29
Navel ill	3	19,293	107.18	0.03 (0.01, 0.08)	2.66
Sink diseases	7	19,265	107.02	0.06 (0.003, 0.13)	6.29
Miscellaneous	10	19,260	107	0.09 (0.005,0.17)	8.88

^ N= Number of cases, CI= confidence interval^

#### Causes of morbidity and mortality in dairy calves

**Fig. 1:**
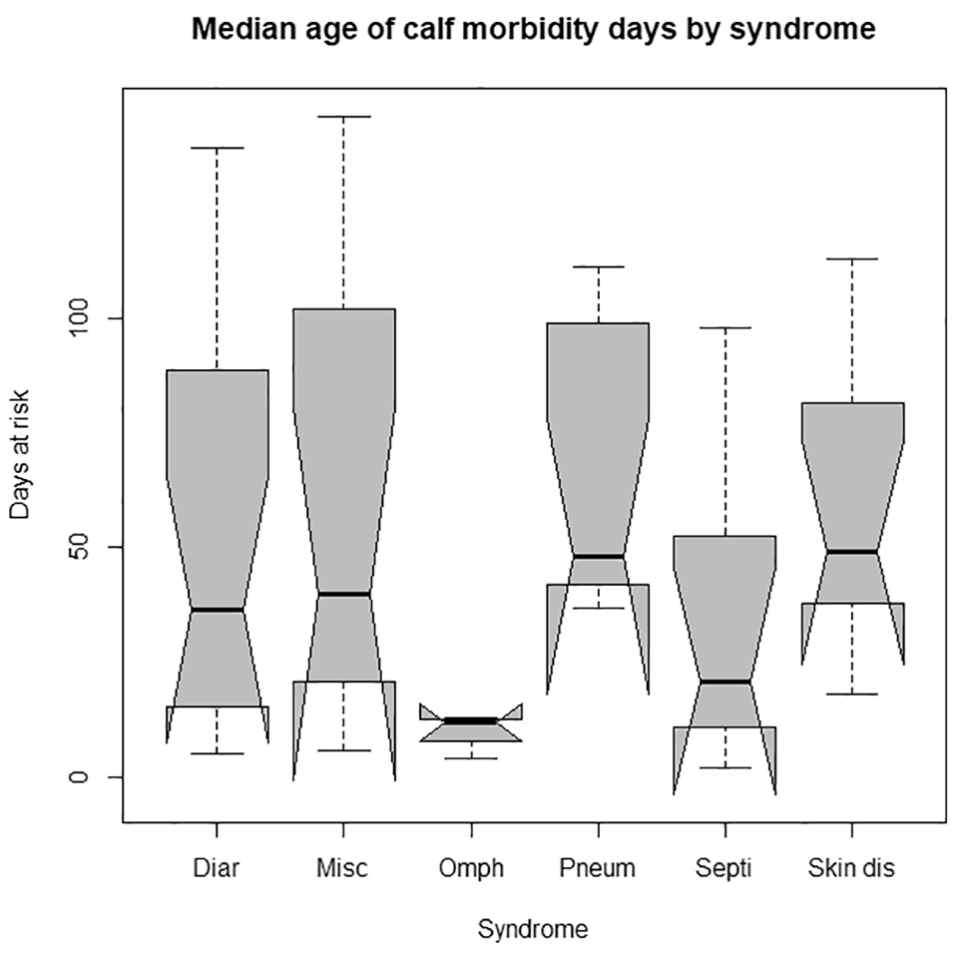
Box-plot of the median age of morbidity by syndromes

Calf diarrhea was the leading cause of morbidity with an incidence rate of 13.84%, followed by pneumonia (8.97%) and septicemia (6.29%) (Table 2). The median time to event analysis of diarrhea, miscellaneous, pneumonia and septicemia, navel ill, and skin disease was 36.5, 40, 48, 21, 13, and 49 days, respectively (Fig. 1). The major cause of mortality was diarrhea (33.33%) followed by pneumonia (26.67%), septicemia (20%), and miscellaneous causes (20%). The median time to death caused by diarrhea, miscellaneous, pneumonia, and septicemia were 28, 107, 49, and 18 days, respectively.

#### Cumulative incidence of morbidity and mortality

**Fig. 2:**
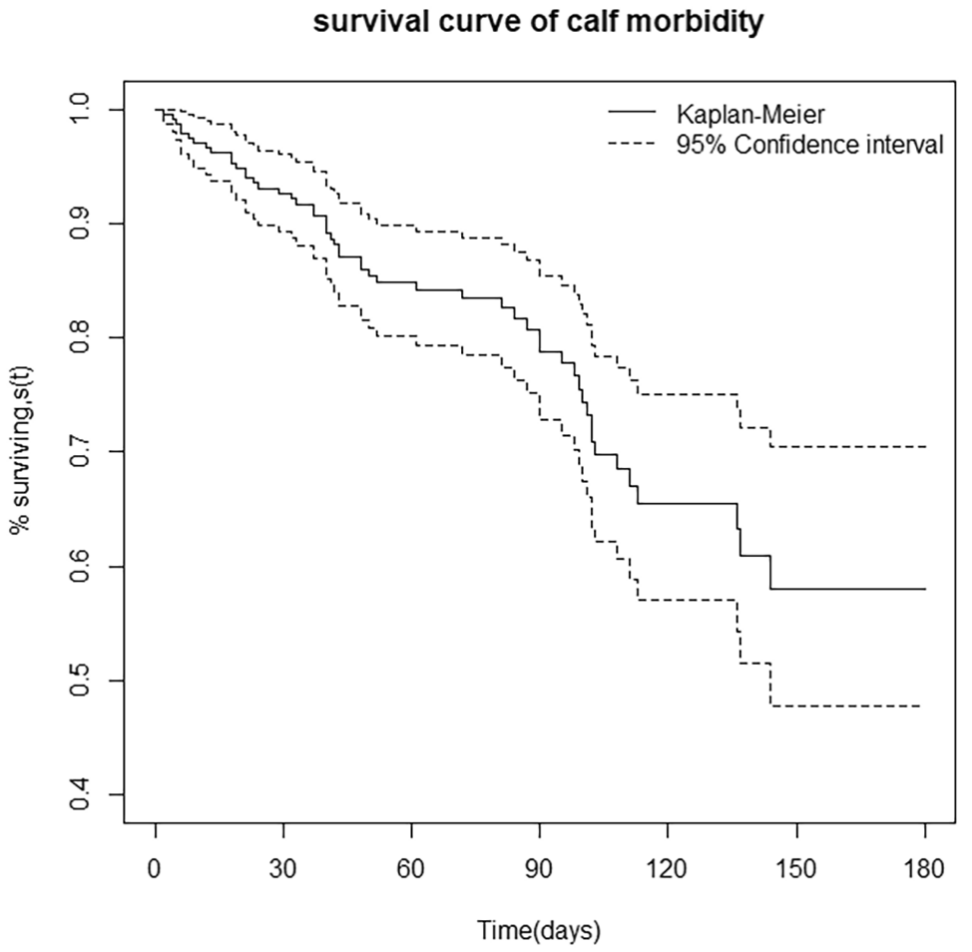
Kaplan-Meier estimates of survivor function of calf morbidity

**Fig. 3:**
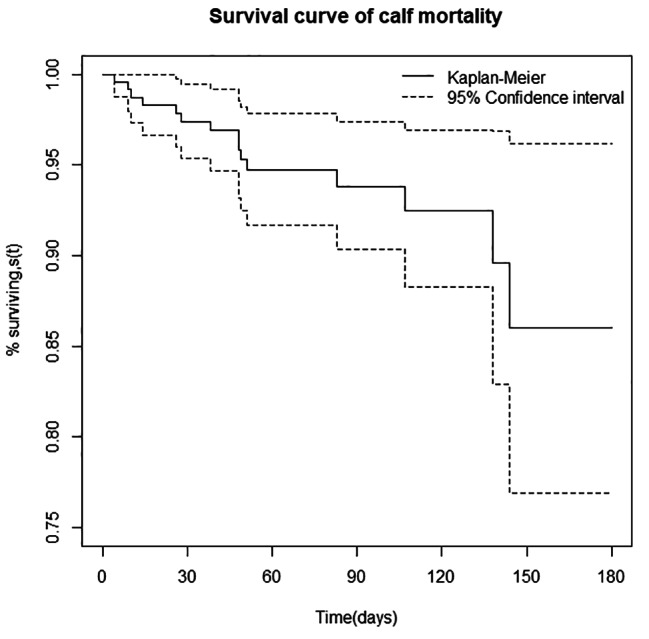
Kaplan-Meier estimates of survivor function of calf mortality

The cumulative survival probability or incidence of morbidity and mortality in the first six months was estimated using the K-M life table as presented in Table 3. The likelihood of a calf getting a disease by the end of the study was 42% (95% CI: 0.03–0.53), while the chance of dying was 12% (95% CI: 0.06–0.25) (Table 3). This suggests the probability of a calf remaining disease-free or surviving at the end of the follow-up period was 58% and 88%, respectively. The K-M curve plot was displayed to show the cumulative survival probability of calves from birth to six months of life (Figs. 2 and 3). In addition, the K-M method was used to compute the hazard of morbidity and mortality for each month of age category. The risks of morbidity and mortality incidence steadily increased in the first four months of the calves’ age while the risk stays constant thereafter up to 6 months old. Interestingly, the greatest risks of morbidity was encountered on the fourth months of age (Table 3).

**Table 3:** Age-specific cumulative survival and incidence of all-cause morbidity and mortality in calves under six months in Jimma town

Age interval (days)	Number at risk	Events	Number censored		Survival prob. (SE)	Hazard (95%CI)	Cum. Incidence (95% CI)
**Morbidity**							
0–30	235	17	20	0.92 (0.02)		0.07(0.04, 0.11)	0.07(0.04,0.11)
30–60	198	15	54	0.84 (0.03)		0.09(0.04, 0.12)	0.15(0.11,0.20)
60–90	129	5	42	0.80 (0.03)		0.04(0.01,0.09)	0.21(0.15,0.28)
90–120	82	13	29	0.65 (0.05)		0.16(0.08,0.27)	0.34(0.26,0.43)
120–150	40	3	21	0.58 (0.05)		0.07(0.01,0.22)	0.42(0.30,0.53)
150–180	16	0	14	0.58 (0.05)		0.00(0.00,0.23)	0.42(0.30,0.53)
180-	2	0	2	0.58 (0.05)		0.00(0.00,0.23)	0.42(0.03,0.53)
**Mortality**							
0–30	235	6	18		0.97(0.01)	0.02(0.01,0.05)	0.03(0.01,0.05)
30–60	211	5	58		0.95(0.02)	0.03(0.01,0.05)	0.05(0.03,0.09)
60–90	148	1	53		0.94(0.02)	0.01(0.00,0.04)	0.06(0.03,0.10)
90–120	94	1	41		0.93(0.02)	0.01(0.00,0.06)	0.07(0.04,0.13)
120–150	52	2	30		0.88(0.04)	0.04(0.005,0.14)	0.12(0.06,0.25)
150–180	20	0	18		0.88(0.04)	0.00(0.00,0.18)	0.12(0.06,0.25)
180-	2	0	2		0.88(0.04)	0.00(0.00,1.84)	0.12(0.06,0.25)

^SE= standard error of estimate, CI= confidence interval^

### Risk factors for calf morbidity and mortality

#### Univariable analysis

A total of 21 calf and herd-level risk factors were tested for a significant relationship with calf morbidity and mortality. Fourteen (14) variables were found to have a P-value < 0.25 with calf morbidity and were subjected to mixed-effect multivariable Cox regression analysis. Whereas, 13 variables have a significant level of < 0.25 with calf mortality (Tables 4 and 5).

**Table 4:** Univariate mixed-effect Cox proportional hazard regression analysis of explanatory variables associated with calf morbidity under six months of age in Jimma town

Variables	Levels	HR (95%CI)	P -value
Dam parity	Multiparous	1	< 0.001
	Primiparous	5.98 (3.48–10.29)	
Calving status	Normal	1	< 0.001
	Dystocia	8.19(4.65–14.44)	
Post-delivery complications	no	1	0.142
	Yes	1.65(0.85–3.20)	
Source of income	Primary	1	0.201
	secondary	1.44(0.82–2.50)	
Experience (year)	≤ 5 year	1	0.004
	> 5 yr	0.45(0.26–0.78)	
Herd size	> 20	1	0.032
	≤ 20	0.51(0.28–0.94)	
Floor type	Concrete	1	< 0.001
	Soil	4.31(2.50–7.43)	
Calving pen	not-present	1	0.232
	present	0.62(0.28–1.36)	
Calf housing	With dam	1	0.033
	Separate	0.51(0.27–0.95)	
Pen cleaning	Infrequently	1	< 0.001
	Regularly	0.11(0.06–0.20)	
Pen Bedding	Absent	1	0.130
	Present	0.62(0.34–1.14)	
1st colostrum feeding	Within 6 h.	1	< 0.001
	After 6 h.	6.70(3.86–11.63)	
Milk-fed daily	< 3 lit.	1	0.055
	> 3lit.	0.52(0.27–1.01)	
Water provision	Periodic	1	0.239
	Free	0.54(0.19–1.50)	

^HR*= Hazard ration, CI= confidence interval, yr.= year, hr.= hour^

**Table 5:** Univariate mixed-effect Cox proportional hazard regression analysis of explanatory variables associated with calf mortality under six months of age in Jimma town

Variables	Categories	HR (95%CI)	P -value
Dam parity	Multiparous	1	< 0.001
	Primiparous	11.42 (3.87–33.72)	
Calving status	Normal		< 0.001
	Dystocia	20.58 (6.96–60.88)	
Post-delivery complications	No	1	0.213
	Yes	2.08 (0.66–6.55)	
Experience (year)	≤ 5 year	1	0.002
	> 5 yr	0.13 (0.04–0.47)	
Herd size	> 20	1	0.028
	≤ 20	0.10 (0.01–0.78)	
Floor type	Concrete	1	< 0.001
	Soil	75.17 (9.88-572.05)	
Calving pen	Not-present	1	0.243
	Present	0.30 (0.04–2.27)	
Calf housing	With dam	1	0.077
	Separate	0.26 (0.06–1.16)	
Pen cleaning	Irregularly	1	0.016
	Regularly	0.28 (0.10–0.79)	
Pen bedding	Absent	1	0.220
	Present	0.45(0.13–1.61)	
1st colostrum feeding	Within 6 h.	1	0.095
	After 6 h.	982.79 (0.30-3223682.18)	
Milk-fed daily	< 3 lit.	1	0.059
	> 3lit.	0.14 (0.02–1.07)	
Colostrum feeding	Hand	1	0.231
	Suckling	0.50 (0.16–1.56)	

^HR*= Hazard ration, CI= confidence interval, yr.= year, hr.= hour^

#### Multivariable analysis of calf morbidity

A total of two variables were kept in the final model that has a significant effect on calf morbidity. Accordingly, dam parity (p < 0.001) and calf pen cleaning pattern (p < 0.001) were the predictor variables that remained in the final model (Table 6). The risk of morbidity was higher for calves born to a primiparous dam (HR = 3.24, 95% CI: 1.65–6.36) than born to a multiparous dam, and the risk of morbidity was lower for calves kept in a regularly cleaned house (HR = 0.17, 95% CI: 0.08–0.38).

**Table 6:** Multivariable mixed-effect Cox proportional hazard regression analysis of explanatory variables associated with the incidence of morbidity in calves under six months of age in Jimma town

Variables	Levels	HR (95%CI)	P-value
Dam parity	Multiparous	1	< 0.001
	Primiparous	3.24 (1.65–6.36)	
Pen cleaning	Infrequently	1	< 0.001
	Regularly	0.17 (0.08–0.38)	

^HR*= Hazard ratio, CI= confidence interval, hr.= hour, NA= not applicable^

**Fig. 4:**
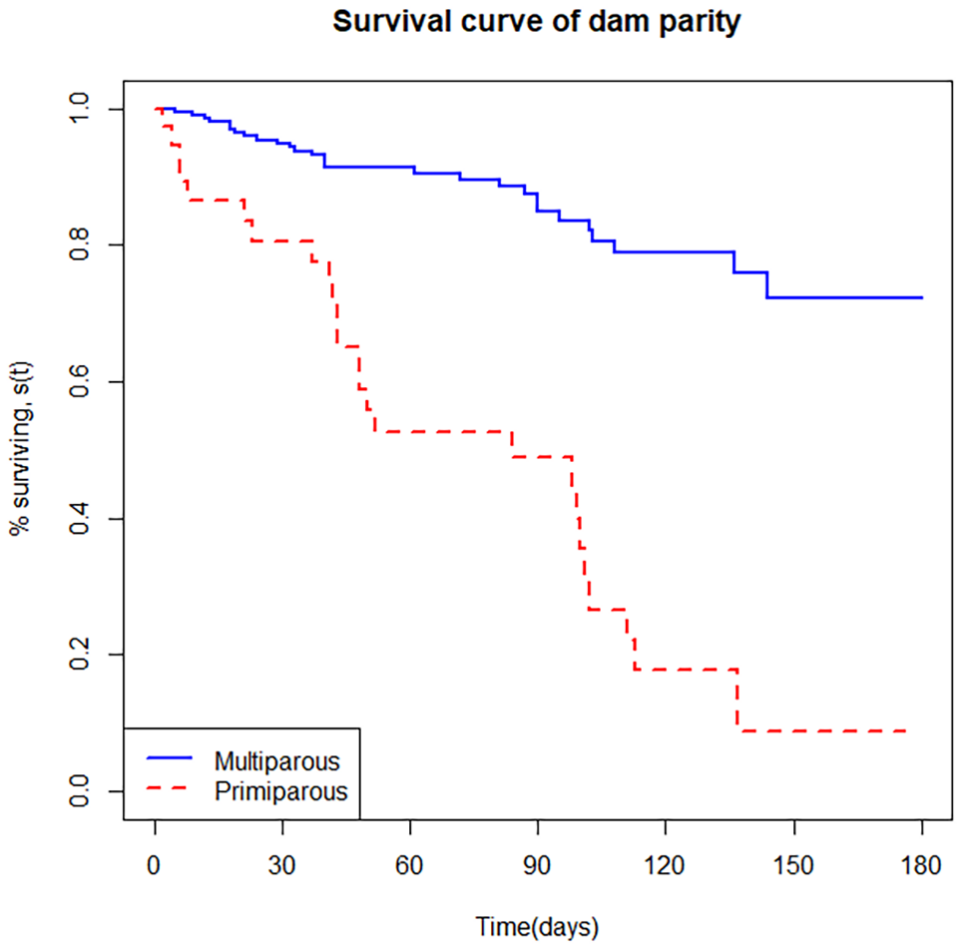
Kaplan-Meier estimates of survivor function for calf morbidity with dam parity strata

**Fig. 5:**
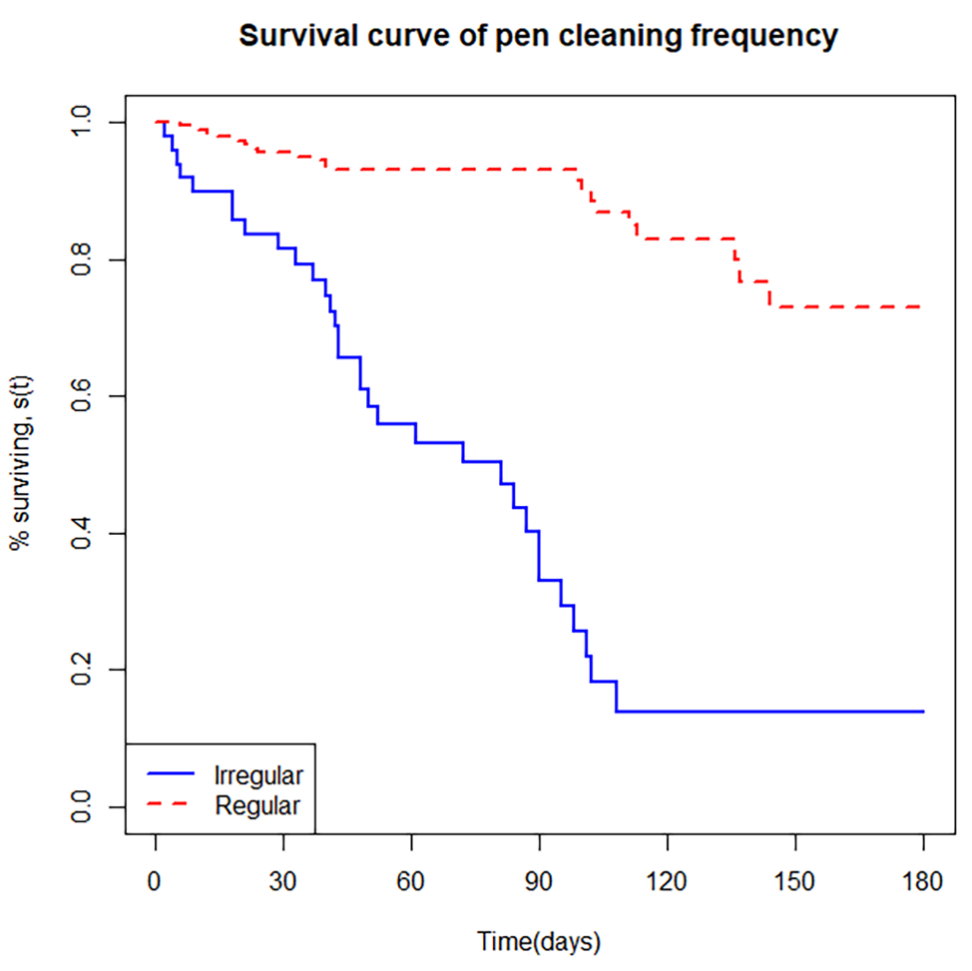
Kaplan-Meier estimates of survivor function for calf morbidity with calf pen cleaning frequency strata

The survival probability plots of calf morbidity for dam parity and calf pen cleaning pattern strata were presented in Figs. 4 and 5. The plots showed that calves born to a primiparous dams and irregularly cleaned calf pens had a smaller survival probability than a multiparous dam and regularly cleaned calf pen, respectively.

#### Multivariable analysis of calf mortality

Four explanatory factors, including dam parity (p = 0.007), calving status (p = 0.005), calf pen cleaning frequency (p = 0.04), and floor type (p = 0.001) have a significant relationship with the incidence of mortality (Table 7). The hazard of mortality was higher for calves born to a primiparous dam (HR = 9.60, 95% CI: 1.86–49.48), assisted delivery (HR = 17.23, 95% CI: 2.41-123.31), and irregularly cleaned house (HR = 7.98, 95% CI: 1.04–60.89) and soiled housing floor type (HR = 153.54, 95% CI: 6.91-3410.85) (Table 7).

**Table 7:** Multivariable mixed-effect Cox proportional hazard regression analysis of explanatory variables associated with the incidence of mortality in calves under six months of age in Jimma town

Variables	Levels	HR (95%CI)	P-value
Dam parity	Multiparous	1	0.007
	Primiparous	9.60 (1.86–49.48)	
Pen cleaning	Regularly	1	0.04
	Infrequently	7.97 (1.04–60.89)	
Calving status	Normal	1	0.005
	Dystocia	17.23 (2.41-123.31)	
Floor type	Concrete	1	0.001
	Soil	153.54 (6.91-3410.85	

^ h*= Hazard ratio, CI= confidence interval, hr.= hour, NA= not applicable^

#### Multivariable analysis of diarrhea and Respiratory Disease as the causes of morbidity and mortality

The hazard for diarrhea was significantly associated with calf sex (p = 0.003), first colostrum feeding time (p = 0.028), breeding methods (p = 0.013), and pen cleaning frequency (p = 0.010). There was a higher risk of diarrhea in male calves (HR = 6.18, 95% CI: 1.85–20.61), calves fed first colostrum 6 h after calving (HR = 5.07, 95% CI: 1.19–21.64), calves born through the natural service breeding method (HR = 13.12, 95% CI: 1.74–99.07). Conversely, calves housed in regularly cleaned pens (HR = 0.15, 95% CI: 0.04–0.64) were less likely to be affected by diarrhea. Calves fed first colostrum 6 h after calving (HR = 11.47, 95% CI: 1.09-120.18) had a higher risk of getting pneumonia than those fed first colostrum within 6 h after calving.

The hazard of mortality due to diarrhea was higher in calves born to a primiparous (HR = 4.20, 95% CI: 1.43–7.04) and male calves (HR = 4.10, 95% CI: 1.23–6.34) than multiparous and female calves, respectively. In addition, calves born to a primiparous dam (HR = 4.16, 95% CI: 1.76–6.87) and feeding colostrum 6 h after calving (HR = 6.37, 95% CI: 3.24–10.27) were more likely to develop pneumonia leading to the death of calves than calves born to a multiparous dam and feeding first colostrum before 6 h after calving.

The final models of morbidity and mortality were tested for the Cox regression model assumption of proportional hazards (PH). Accordingly, the PH assumption for morbidity (χ^2^ = 1.23, P = 0.06) and mortality (χ^2^ = 2.06, P = 0.560) was not violated. Additionally, the survival curves of predictor variables did not cross, which further substantiates the PH assumption is not violated. Thus, we inferred that the proportionality assumption is satisfied and the coefficient estimate of the hazard ratio between groups of the predictors in the final model remained constant over the survival time.

## Discussion

Worldwide, the major problems in the dairy production system are calf morbidity and mortality [25]. Efficient dairy production and limited losses have been thought important for farm producers to accomplish high productivity in dairy farming. The present study aimed to assess the calf management practices, estimate the extent of calf morbidity and mortality incidence and assess its potential risk factors in Jimma City.

The morbidity rates in this study was similar to previous studies conducted in different parts of Ethiopia [9, 13, 26]. However, our findings were different from those in previous studies [7, 16, 27 &^28^].

Furthermore, the mortality rate found in this study (12.97%) was slightly consistent with the findings reported by Megersa et al. [28] and Phiri et al. [29]. However, it was lower than the mortality rates ranging from 17.9 to 30.7% reported by previous authors [7, 13, 16, 26, 30]. Conversely, it is higher than previous reports [5, 27, 31–33]. The K-M survival functions revealed that the probability of a calf remaining disease-free (58%) or surviving (88%) at the end of the follow-up period was consistent with the finding of Abebe et al. [34].

The discrepancy between the present and previous findings in different parts of Ethiopia and elsewhere might be the variations in the length of prospective study, calf, and herd-level risk factors, herd size, breed, management system, and climatic conditions. For instance, most of the prior studies in parts of Ethiopia employed less powerful study designs, such as a cross-sectional study [30] and computed calf morbidity and mortality using prevalence which did not properly show the riskof morbidity [26, 30]. In addition, the decline in calf mortality in dairy production could be linked to improved management practices on dairy farms,, differences in calf age, herd size, breed, and agroecology [16].

The highest risks of morbidity and mortality were in the first four months of the calves’ age while the risk kept constant thereafter up to 6 months old. Surprisingly, the risks of morbidity was higher on the fourth months of age which could be attributed to calves stayed without being introduced to dry feed upto weaning age and no special starter feed was provided. Thus, weaned calves failed to adjust to dry feed quickly and efficiently which leads to malnutrition and thereby suppress immune syetem of the calf.

Diarrhea followed by pneumonia was the most common health issue among calves. Additionally, they were also the leading causes of death. This is consistent with previous research conducted in different parts of Ethiopia and other countries [13, 16, 35, 36]. This might be due to management-related; especially unhygienic conditions in the house and mixing of calf with other adult animals could explain it. The incidence rate of calf diarrhea found in this study is comparable with Rahma et al., [9]. On the contrary, our finding is lower than the findings of Wudu et al. [16]and Ferede et al. [30]. The observed difference could be attributed to factors such as the difference in the quantity of colostrum consumed, the cleanliness of feeding equipment, and state of the calf house [13].

The hazard of morbidity and mortality was higher in calves born to a primiparous cows than multiparous cows. This difference could be attributed to the variation in immunoglobulin (Ig) concentration among cows with different parity levels. Primiparous dams had lesser exposure time to pathogens which resulted in a deficiency of important Ig in their colostrum secretions [16]. The study conducted in China by Liu et al. [37] revealed that the immunoglobulin concentration in a multiparous cow is 1.3–1.6 times greater than in primiparous cows. Similarly, Downey et al. [38] revealed that the maternal antibody transferred to offspring is higher in older than younger dams. In addition, the incidence of dystocia and stillbirth is higher in the first-calving cow [39, 40].

The smaller risk of morbidity in calves housed in regularly cleaned pen found in this study agrees with the reports of Wudu et al. [16], Marce et al. [41] and Assefa and Ashenafi [7]. This suggests that unhygienic calf pens could put the calf in a stressful environment, exposed to an overwhelming pathogen, compromising their immune system, which leads to calf health problems [16, 42].

Calves born to cows requiring assisted delivery (dystocia) had a higher mortality risk than calves born with normal delivery. The current finding is in agreement with Lombard et al. [43] and Asmare and Kiros [7]. Difficult calving leads to delayed onset of colostrum feeding, reduced colostrum intake, and contamination which increases the hazards of morbidity and mortality of calves [44]. Furthermore, stress during parturition leads to the secretion of the adrenocorticotropic hormone that stimulates the adrenal cortex to maximize the production and release of cortisol causing immunosuppression [45]. Gulliksen et al. [40] reported that dystocia is the most common cause of perinatal mortality.

Calves kept on soiled floors had a high risk of death compared to calves kept on concrete floors. This could be attributed to the difficulty of keeping dirty floors clean and dry. In addition, they were also less efficient at disinfection. As reported by Lindsay [46], wet and muddy conditions have proven to be the source of increased morbidity that leads to death because pathogenic bacteria can grow rapidly.

The risk of diarrhea was higher in male than female calves. This could be attributed to the good management and health care offered for female calves on the farms. Female calves are considered future replacement stocks on farms and are of greater economic significance. As a result, male calves often get less attention to feeding, medical care, and others. A similar finding was also been reported by other studies conducted in Ethiopia [28].

Calves that are born through natural service were at higher risk of getting diarrhea than artificial insemination. This could be explained by the infections that cause diarrhea spreading from the bull to the cow and calf, such as bovine viral diarrhea. However, our finding contradicts the reports of Medeiros et al. [47] who reported diarrhea was more frequent in herds that use artificial insemination (AI).

The time at 1st colostrum feeding is among the risk factors significantly related (p = 0.028) to diarrhea syndrome. A greater hazard of diarrhea among calves that start to feed the first colostrum six hours after birth compared to calves fed colostrum within 6 h after birth. Similar findings were reported by Phiri [33], Ferede et al. [30], Wudu et al. [16], and Tora et al. [27]. This could be due to the inability of the calves to get sufficient and fresh uncontaminated colostrum directly from their dams and could also be the loss of passive immunity in the colostrum resulting from improper storage and contamination. According to Moran [2], there is a 10% increment in the likelihood of a calf getting sick for every hour delay to feed colostrum in the first 12 h of early life. Because the concentration of immunoglobulin (IgG) found in colostrum and its absorption from the small intestine reduces over time [48]. Consequently, the calf is vulnerable to contracting harmful pathogens and diseases since the calf lacks an immune system. Thus, adequate colostrum needs to be provided to obtain ready-made antibodies for calves to boost their immune system [49, 50&^51^].

### Study limitations

This study has potential limitations. Firstly, the limited sample size of the study could influence the precision of estimates and the power of the study. Additionally, the small number of calves/farms could affect the statistical power of the test. Secondly, the purposive sampling technique employed for recruiting study animals and farms could face sampling bias, and the sample was not representative of the entire population. Lastly, the presence of a small number of calves under the soiled floor level of floor type (soiled vs. concrete) in the calf mortality model led to a large hazard ratio estimate and a wider confidence interval. Finally, this study doesn’t consider the identification of causative agents of the morbidity and mortality of a calf. As a result, we couldn’t associate the causative agent with calf morbidity and mortality.

## Conclusions

This study aims to assess the management practices, morbidity, and mortality incidence of a calf and its associated determinants in dairy calves in Jimma City, Ethiopia. The cumulative incidence of morbidity and mortality in calves in the first six months of age was found to be 42% and 12%, respectively. In addition, the major causes of morbidity and mortality were diarrhea, pneumonia, and septicemia. The dam parity, calf pen cleaning frequency, floor type, and calving status were found to have a significant effect (p *<* 0.05) on the morbidity and mortality of calves. As a result, calves born to a primiparous dam, grow in less frequently cleaned pens, kept on soiled floor, and calves a born to assisted delivery were more likely to be sick and or die from different diseases. The mortality incidence rate found surpassed the economically acceptable limit that could be attained through adopting good management practices. Thus, proper intervention in the management practice of calves is required.

### Electronic supplementary material

Below is the link to the electronic supplementary material.

Supplementary Material 1

Supplementary Material 2

Supplementary Material 3

Supplementary Material 4

## Data Availability

The datasets generated and/or analyzed during the current study are available in the figshare repository, 10.6084/m9.figshare.19519828.v1.
