# Supplementary material for: Calf morbidity, mortality, and management practices in dairy farms in Jimma City, Southwestern Ethiopia
Source: BMC Vet Res. 2023 Nov 28;19:249. doi: 10.1186/s12917-023-03815-w (PMC10683357; doi:10.1186/s12917-023-03815-w)
Supplement: Supplementary file 1 — Supplementary Material 1 [file 12917_2023_3815_MOESM1_ESM.docx]

Supplementary file 1: Sample questionnaire for collection of herd level data

**1. General information**

Date of interview_________/_______/_______

Farm name_________________________________________________________

Name of farm owner_________________________________________________

Address__________________________ Phone number______________________________

**2. Farm owner/manager attributes**

2.1. Sex a) Male b) Female

2.2. Educational status a) No formal education b) Primary education c) High school education d) College or university graduate

2.3. Dairy production as a source of income a) Primary b) Secondary

2.4. Dairy farming experience a) < 5 yr b) > 5yr

**3. Farm characteristics and managemental practice**

3.1. Type of managemental practices a) Intensive b) Semi intensive

3.2. Herd size and composition

| Herd composition | | Local | Cross | Total |
| --- | --- | --- | --- | --- |
| Calves | Female |  |  |  |
|  | Male |  |  |  |
| Heifers | |  |  |  |
| Lactating cow | |  |  |  |
| Dry cow | |  |  |  |
| Bull | |  |  |  |
| Total herd size | |  |  |  |

3.3. Breeding method a) Artificial insemination b) Natural mating/bull service

3.4. House type a) Concrete b) Non concrete

3.5. Floor type a) Concrete b) Soil

3.6. House cleaning frequency a) Regularly b) Infrequently

If regularly, how many times a day?

a) Once b) Twice a day c) Three times a day

**4. Dry cow management and periparturient care**

4.1. Do you provide dry period to your cow? a) Yes b) No

If yes, how long is the period? a) < 8 wk b) >8 wk

4.3. Do you Provide vitamins and minerals for pregnant cow? a) Yes b) No

If yes, what types of vitamin and mineral do you provided?

4.4. Do you vaccinate and provide preventive treatment to pregnant cow? a) Yes b) No

4.5. Do you have calving pen? a) Yes b) No

If yes:

Location a) Cow barn b) Calf barn c) Separate outside

Cleaning before calving a) Yes b) Sometimes if dirty c) No

Bedding material a) Straw b) Stalk c) Sawdust d) No

4.6. Do you provide calving assistance a) Yes b) No

If yes, when do you provide calving assistance? __________________________________

If yes, by whom? ___________________________________________________________

4.7. Drying of the calf a) By dam b) Artificially

4.8. Do you disinfecting calf’s navel/umbilicus? a) Yes b) No

If yes, what type of chemical do you use? _________________________________________

4.9. When you separate calf from dam? ___________________________________________

**5. Colostrum management**

5.1. Do you provide colostrum to calf? a) Yes b) No

5.2. Is colostrum important to calf? a) Yes b) No

5.3. Time of first colostrum feeding a) <6 hr b) 6-24 hr c) > 24 hr

5.4. Amount of first colostrum feed a) <1L b) 1-1.5L c) >2 e) Unknown

5.5. Duration of colostrum feeding a) <24 hr b) 24 hr-4 days c) >4 days

5.6. Method of feeding colostrum a) Suckling b) Bucket/hand feeding

**6. Calf housing**

6.1. Location a) In separate house b) With dam

6.2. Pen type a) Individual pen b) Group pen

If group pen, how may calves kept per pen? ______________________________

6.3. Bedding material a) Straw b) Sawdust c) Stalk d) Other e) No

**7. Provision of milk, dry feed and water**

7.1. Source of milk a) Dam milk b) Pol milk c) Milk replacer

7.2. Frequency of feeding milk/day a) Once b) Twice a day c) Three times a day

7.3. Maximum amount of milk given per day a) <3 L b) >3 L

7.4. Do you provide feed other than milk or milk replacer? a) Yes b) No

If yes, when you introduce additional feed? a) > wk b) 1-2 wk c >2 wk

If yes, Could you mention major types of feeds you are provided?

7.5. Water provision a) Free access b) Periodic

7.6. Source of water a) Pipe line b) River c) Ground water

7.8. Weaning age a) < 6 wk b) 6- 8 wk c) > 8 wk

**8. Health care and problem**

8.1. Measures to treat sick calf

a) Calling animal health professional b) Take to a nearby vet clinic c) Have employed animal health professional

8.2. Is calf morbidity is problem of your farm? a) Yes b) No

8.3 Is calf mortality is the problem of your farm? a) Yes b) No

8.4. Total number of calves (< 6month) the farm lost during the last one year

Local ____________ Cross ___________Total______________
