# Supplementary material for: Calf morbidity, mortality, and management practices in dairy farms in Jimma City, Southwestern Ethiopia
Source: BMC Vet Res. 2023 Nov 28;19:249. doi: 10.1186/s12917-023-03815-w (PMC10683357; doi:10.1186/s12917-023-03815-w)
Supplement: Supplementary file 2 — Supplementary Material 2 [file 12917_2023_3815_MOESM2_ESM.docx]

Supplementary file 2: Standardized case definitions of morbidity and mortality events

| No | Disease condition | Case definition |
| --- | --- | --- |
| 1 | Diarrhea | Manure is of looser consistency than normal calves. Any condition characterized by passing of lose or watery feces with increased frequency, which could or could not be accompanied by other systemic signs like dehydration, decreased appetite or fever |
| 2 | Pneumonia | Increased resting respiratory rate, fever (>39.5^0^c) with one or more additional signs such as coughing, nasal discharge, depression, decreased appetite or rough hair coat |
| 3 | Septicaemia | Any condition characterized by depression, anorexia and fever without any distinct involvement of specific body system |
| 4 | Naval ill/Omphalitis | Warm, enlargement and pain full umblical cord, or foul smelling discharge from the umblical structures due to infection |
| 5 | Skin diseases | Characterized by skin lesion, scratching, hair loss, presence of ecto- parasite, fever, and lymphadenopathy. |
| 6 | Miscellaneous cases | Different health problems that could not be grouped in any one of the other groups mentioned before and diagnosed relatively less frequently Heart water, Traumatic injury, bloat, eye infection, bloat, ruminal acidosis |
| 7 | Morbidity | Morbidity is defined as any sickness with recognizable clinical signs which ultimately ended in death or warranted therapeutic intervention during the course of follow up period. |
| 8 | Mortality | Mortality is defined as any observed death irrespective of cause. |

**Source:** (Heinriches and Radostitis, 2001; Wudu, 2004; Windyer *et al*., 2014; Yeshwas, 2015; Rahman *et al*., 2020)
